# Consolidated bioprocessing of lignocellulose for production of glucaric acid by an artificial microbial consortium

**DOI:** 10.1186/s13068-021-01961-7

**Published:** 2021-04-30

**Authors:** Chaofeng Li, Xiaofeng Lin, Xing Ling, Shuo Li, Hao Fang

**Affiliations:** 1 College of Life Sciences, Northwest A&F University, 22 Xinong Road, Yangling, Xianyang, 712100 Shaanxi China; 2 Biomass Energy Center for Arid and Semi-Arid Lands, Northwest A&F University, 22 Xinong Road, Yangling, Xianyang, 712100 Shaanxi China

**Keywords:** Consolidated bioprocessing (CBP), d-Glucaric acid, Lignocellulose, Microbial consortium, *Trichoderma reesei*, *Saccharomyces cerevisiae*

## Abstract

**Background:**

The biomanufacturing of d-glucaric acid has attracted increasing interest because it is one of the top value-added chemicals produced from biomass. *Saccharomyces cerevisiae* is regarded as an excellent host for d-glucaric acid production.

**Results:**

The *opi1* gene was knocked out because of its negative regulation on *myo*-inositol synthesis, which is the limiting step of d-glucaric acid production by *S. cerevisiae*. We then constructed the biosynthesis pathway of d-glucaric acid in *S. cerevisiae* INVSc1 *opi1Δ* and obtained two engineered strains, LGA-1 and LGA-C, producing record-breaking titers of d-glucaric acid: 9.53 ± 0.46 g/L and 11.21 ± 0.63 g/L d-glucaric acid from 30 g/L glucose and 10.8 g/L *myo*-inositol in fed-batch fermentation mode, respectively. However, LGA-1 was preferable because of its genetic stability and its superior performance in practical applications. There have been no reports on d-glucaric acid production from lignocellulose. Therefore, the biorefinery processes, including separated hydrolysis and fermentation (SHF), simultaneous saccharification and fermentation (SSF) and consolidated bioprocessing (CBP) were investigated and compared. CBP using an artificial microbial consortium composed of *Trichoderma reesei* (*T. reesei*) Rut-C30 and *S. cerevisiae* LGA-1 was found to have relatively high d-glucaric acid titers and yields after 7 d of fermentation, 0.54 ± 0.12 g/L d-glucaric acid from 15 g/L Avicel and 0.45 ± 0.06 g/L d-glucaric acid from 15 g/L steam-exploded corn stover (SECS), respectively. In an attempt to design the microbial consortium for more efficient CBP, the team consisting of *T. reesei* Rut-C30 and *S. cerevisiae* LGA-1 was found to be the best, with excellent work distribution and collaboration.

**Conclusions:**

Two engineered *S. cerevisiae* strains, LGA-1 and LGA-C, with high titers of d-glucaric acid were obtained. This indicated that *S. cerevisiae* INVSc1 is an excellent host for d-glucaric acid production. Lignocellulose is a preferable substrate over *myo*-inositol. SHF, SSF, and CBP were studied, and CBP using an artificial microbial consortium of *T. reesei* Rut-C30 and *S. cerevisiae* LGA-1 was found to be promising because of its relatively high titer and yield. *T. reesei* Rut-C30 and *S. cerevisiae* LGA-1were proven to be the best teammates for CBP. Further work should be done to improve the efficiency of this microbial consortium for d-glucaric acid production from lignocellulose.

**Supplementary Information:**

The online version contains supplementary material available at 10.1186/s13068-021-01961-7.

## Background

d-Glucaric acid, identified as a “top value-added chemical from biomass” by the United States Department of Energy in 2004 [1], is an important platform chemical with a wide variety of applications such as therapeutic uses and biopolymer production [2–4]. Conventionally, d-glucaric acid is produced via nitric acid oxidation of d-glucose. This is a nonselective and expensive process associated with a large exotherm, low yields and toxic byproducts [4–6]. Biological production of d-glucaric acid has attracted increased interest due to its potential as a cheaper and more environmentally friendly process that avoids costly catalysts and harsh reaction conditions [3, 4].

A biosynthesis route from d-glucose to d-glucaric acid involving three heterologous genes was constructed in recombinant *Escherichia coli* by Moon et al. [2]. The genes were *myo*-inositol-1-phosphate synthase (Ino1) from *Saccharomyces cerevisiae*, *myo*-inositol oxygenase (MIOX) from *Mus musculus*, and uronate dehydrogenase (UDH) from *Pseudomonas syringae*. However, the titer of d-glucaric acid was low, only 0.72 g/L [2]. After the induction and culture conditions were optimized, a slight increase in the d-glucaric acid titer to 1.13 g/L was achieved [2, 4]. MIOX was found to be the rate-limiting step of the biosynthesis route for such low titers [2]. Hence, a synthetic scaffold was used to increase MIOX stability and the efficiency of the biosynthesis route, leading to a titer of 2.5 g/L d-glucaric acid from 10 g/L d-glucose [7]. Moreover, an N-terminal small ubiquitin-related modifier (SUMO) fusion to MIOX gave rise to a 75% increase in d-glucaric acid production from *myo*-inositol. Up to 4.85 g/L of d-glucaric acid was produced from 10.8 g/L *myo*-inositol in recombinant *E. coli* [4]. However, *E. coli* is thought to be unsuitable for d-glucaric acid production at high titers because d-glucaric acid concentrations above 5 g/L appear to inhibit *E. coli* through a pH-mediated effect [2, 3, 5].

Gupta et al. [5] ported the synthetic d-glucaric acid pathway from *E. coli* to *S. cerevisiae*, another model strain widely used industry that has better acid tolerance [3]. They found that MIOX4 from *Arabidopsis thaliana* outperformed MIOX from *M. musculus* [5]. The maximal d-glucaric acid titer of the *S. cerevisiae* strain with MIOX4 was 1.6 g/L from glucose supplemented with *myo*-inositol [5]. Chen et al. [3] adopted a delta-sequence-based integrative expression to increase the MIOX4 activity and stability, successfully increasing the d-glucaric acid titer to about eight times that of episomal expression. Combining this strategy with fed-batch fermentation supplemented with 60 mM (10.8 g/L) *myo*-inositol, a titer of 6 g/L (28.6 mM) d-glucaric acid was achieved. This was the highest titer for *S. cerevisiae* [3].

In this study, we used the same genes and strategy as reported by Chen et al. [3] to construct the biosynthesis route for d-glucaric acid production in a different baker’s yeast strain, *S. cerevisiae* INVSc1. In light of the finding that *myo*-inositol availability was the rate-limiting step in the *S. cerevisiae* strain with the *miox4* gene from *A. thaliana* [3, 5], the *opi1* gene in *S. cerevisiae* INVSc1 was also knocked out accordingly to remove its negative regulation on *myo*-inositol synthesis [3, 8]. As a result, a more robust engineered strain of *S. cerevisiae* INVSc1 was obtained, producing a record-breaking titer of d-glucaric acid in *S. cerevisiae*. As the top value-added chemical from biomass, however, there have been no reports on d-glucaric acid production from lignocellulose in a biorefinery scenario.

**Fig. 1:**
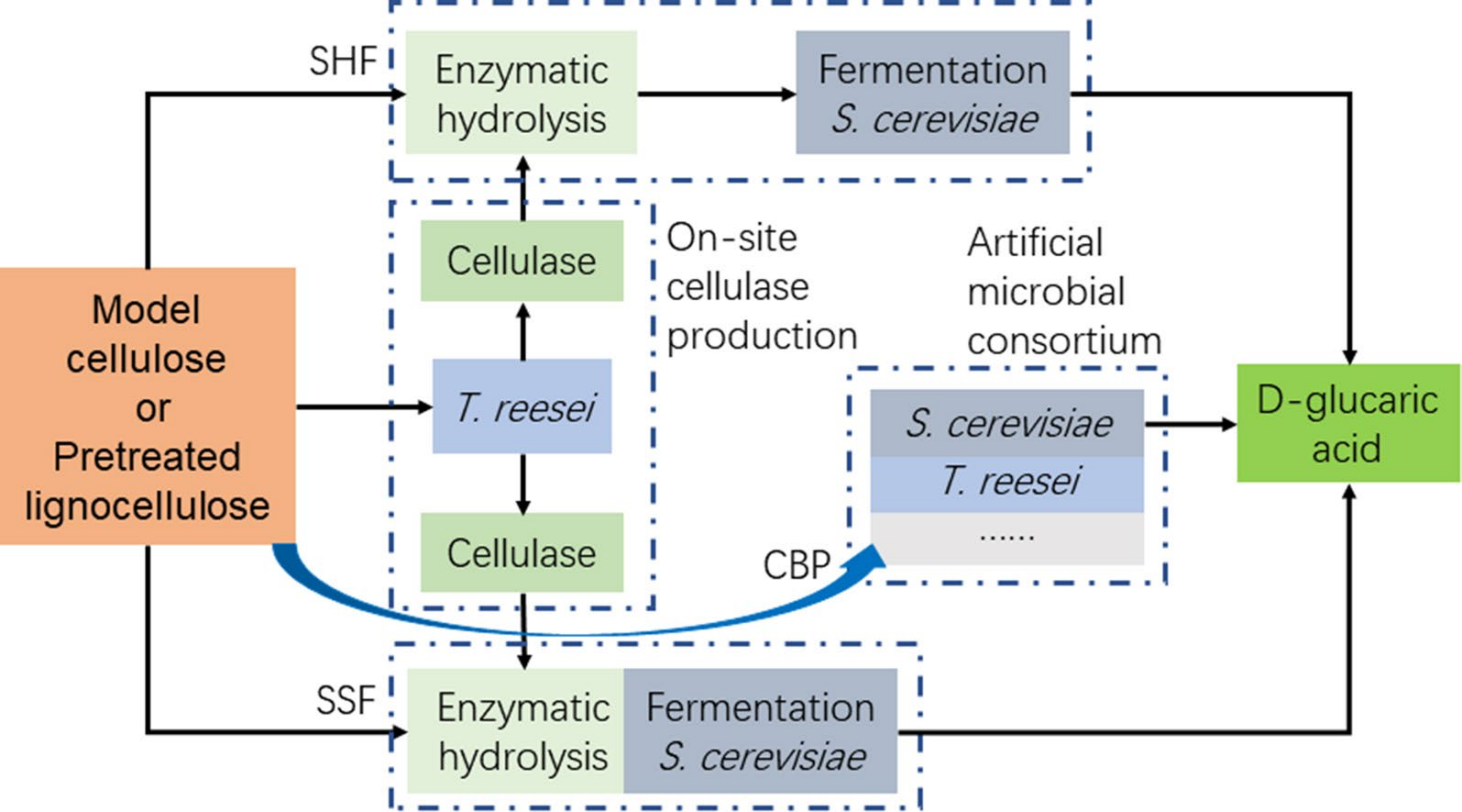
Diagram of biotechnological d-glucaric acid production from lignocellulose. SHF: separated hydrolysis and fermentation; SSF: simultaneous saccharification and fermentation; CBP: consolidated bioprocessing

We applied the engineered *S. cerevisiae* strain to bioprocesses for d-glucaric acid production from model cellulose and natural lignocelluloses. Figure 1 illustrates the biorefinery processes, including separated hydrolysis and fermentation (SHF), simultaneous saccharification and fermentation (SSF), and consolidated bioprocessing (CBP) [9–14]. SHF and SSF were carried out in the context of on-site cellulase production because this mode has many advantages, such as enabling cost savings and using tailor-made enzymes for a given feedstock [13–15]. CBP using an artificial microbial consortium composed of *Trichoderma reesei* Rut-C30 and the engineered *S. cerevisiae* strain was established for direct production of d-glucaric acid from lignocelluloses. Unlike the CBP using the similar microbial consortium for bioethanol production where the fermentation by *S. cerevisiae* was anaerobic but the cellulase production by *T. reesei* was aerobic [12], the CBP in this work was simpler because both the cellulase production by *T. reesei* and d-glucaric acid biosynthesis by *S. cerevisiae* were aerobic. Though artificial microbial consortia were redesigned for more efficient CBP, the team of the two strains was still the best, achieving d-glucaric acid production from lignocelluloses at a near gram per liter level. This work provides an example of d-glucaric acid production at a record-breaking titer and presents the discovery that the CBP of lignocellulose using an artificial microbial consortium is a desirable and promising approach for d-glucaric acid production.

## Results

### Construction of d-glucaric acid-producing ***S. cerevisiae***

**Fig. 2:**
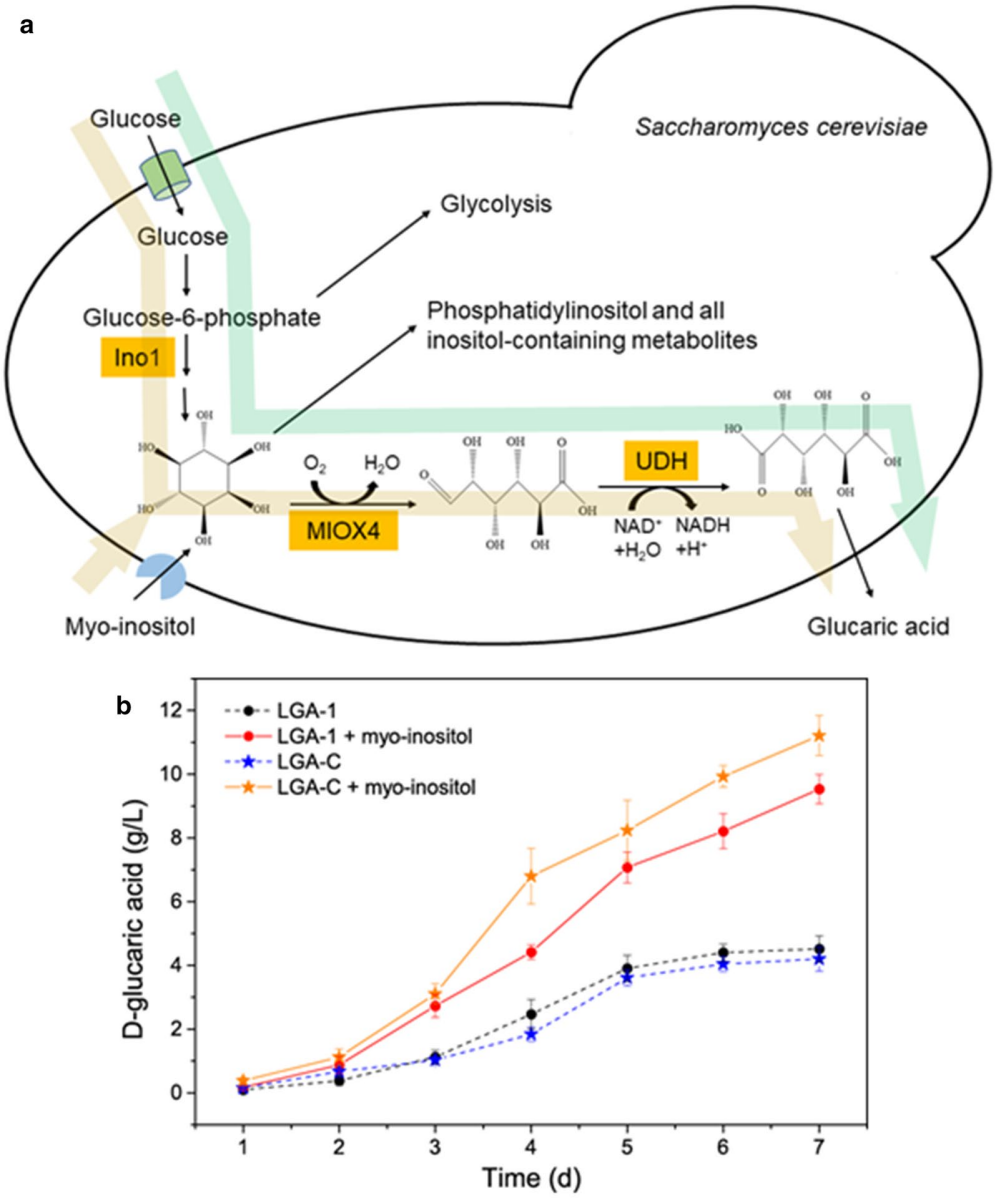
**a** Biosynthesis pathway of d-glucaric acid introduced into *S. cerevisiae*. Ino1: *myo*-inositol-1-phosphate synthase; MIOX4: *myo*-inositol oxygenase; UDH: uronate dehydrogenase. The green arrow shows the d-glucaric acid production from glucose, and the yellow arrow shows d-glucaric acid production from glucose and *myo*-inositol. **b** Time courses of fed-batch fermentations in yeast extract peptone (YPD) medium without and with 10.8 g/L *myo*-inositol. LGA-1 and LGA-C are the engineered *S. cerevisiae* strains capable of producing d-glucaric acid. The data shown here are average values of at least three biological replicates, and the error bars represent standard deviations

It has been reported that the availability of *myo*-inositol was the limiting step for d-glucaric acid in *S. cerevisiae* [3, 5]. The synthesis of *myo*-inositol is negatively regulated by *opi1* [8, 16]. To relieve the negative regulation, therefore, *opi1* was knocked out to generate the *S. cerevisiae* strain INVSc1 *opi1*Δ [3, 5], which was subsequently used as the starting strain for the construction of d-glucaric acid-producing strains. The biosynthesis pathway of d-glucaric acid in the engineered *S. cerevisiae* is illustrated in Fig. 2a. It was also found that the deletion of *opi1* promoted d-glucaric acid production (data not shown), which was in line with a previous report [3]. Two d-glucaric acid-producing strains, *S. cerevisiae* LGA-1, with the integrative expression of foreign genes, and *S. cerevisiae* LGA-C, with the episomal expression, were screened and used for subsequent fermentation experiments. The products of *S. cerevisiae* LGA-1 and LGA-C were characterized by liquid chromatography–mass spectrometry (LC–MS) to verify their capability for producing d-glucaric acid, and the results were confirmative, as shown in Additional file 1: Fig. S1.

### Fed-batch fermentation with or without *myo*-inositol

These two strains, *S. cerevisiae* LGA-1 and *S. cerevisiae* LGA-C, were compared in terms of fermentation in yeast extract peptone dextrose (YPD) medium without and with 10.8 g/L (60 mM) *myo*-inositol [3, 5]. The time courses of the fermentations are shown in Fig. 2b. The fermentation processes supplemented with *myo*-inositol had a higher d-glucaric acid titer than those using glucose as the sole carbon source. This observation was the same as that in previous reports [3, 5]. LGA-1 performed similarly to LGA-C when using glucose as the sole carbon source, in contrast to their performances in fermentation supplemented with *myo*-inositol, where LGA-C outperformed LGA-1. After 7 d of fermentation, LGA-1 produced 9.53 ± 0.46 g/L and LGA-C produced 11.21 ± 0.63 g/L d-glucaric acid from 30 g/L glucose and 10.8 g/L *myo*-inositol in fed-batch fermentation mode.

Production of d-glucaric acid from *myo*-inositol (marked with yellow arrows in Fig. 2a), however, is not a desirable approach because the latter is also an industrially value-added chemical [17, 18]. Moreover, there have not been any reports pertaining to d-glucaric acid production from lignocellulose, though it is considered to be one of the top value-added chemicals from biomass [1]. Therefore, production of d-glucaric acid from lignocellulose in the context of a biorefinery, similar to the production of lignocellulosic biofuels such as lignocellulosic ethanol [9, 10], should be treated as an important attempt to seek cheaper biomanufacturing of d-glucaric acid. Hence, LGA-C and LGA-1 were applied to the d-glucaric acid production from Avicel and pretreated corn stover via SHF, SSF, and CBP, which are typical bioprocesses in a biorefinery.

### Separated hydrolysis and fermentation (SHF)

**Fig. 3:**
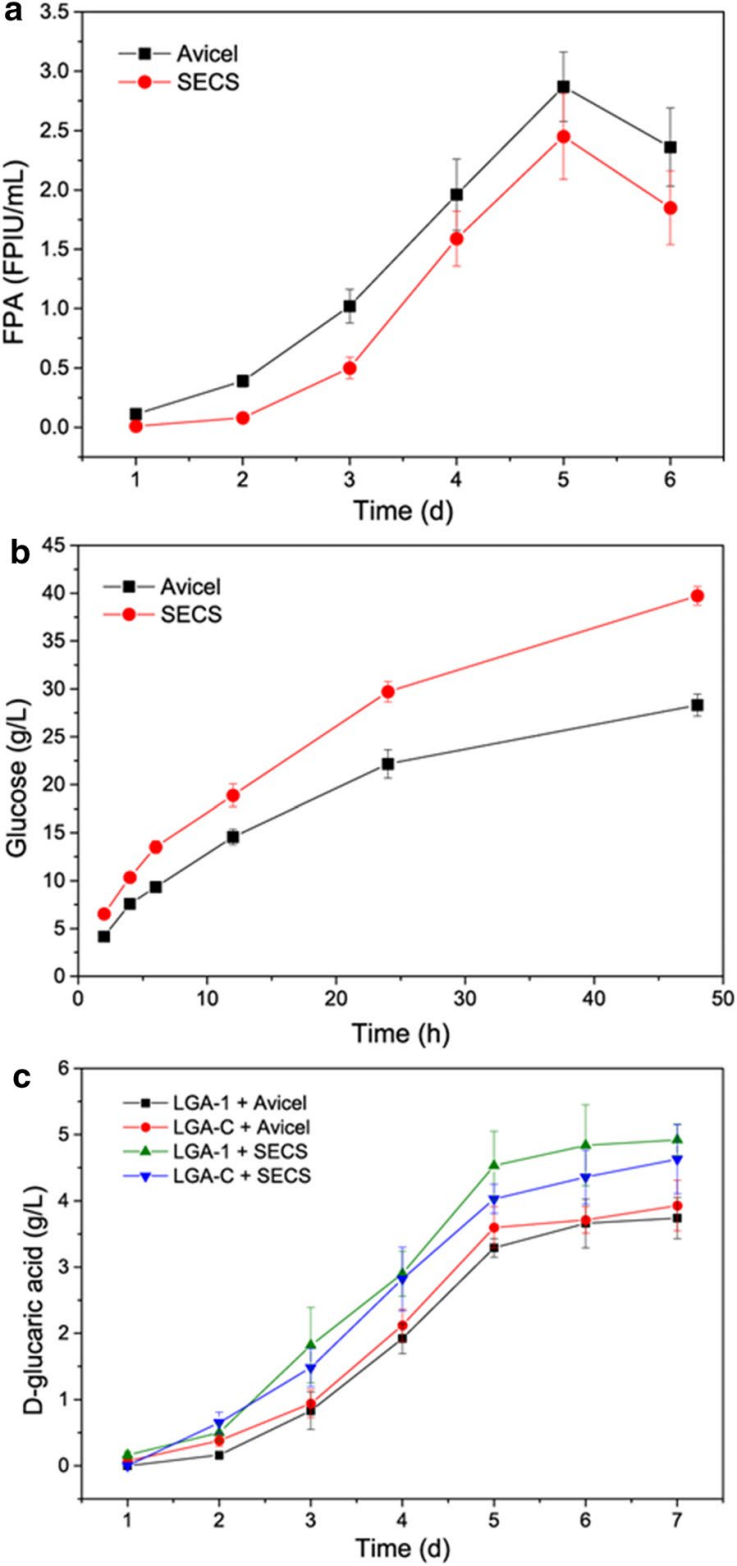
**a** Time courses of cellulase production by *T. reesei* Rut-C30 from Avicel and steam-exploded corn stover (SECS). **b** Enzymatic hydrolysis of Avicel and SECS by the cellulases produced in the context of on-site cellulase production. **c** Time courses of batch fermentations on Avicel hydrolysate and SECS hydrolysate. LGA-1 and LGA-C are the engineered *S. cerevisiae* strains capable of producing d-glucaric acid. The data shown here are average values of at least three biological replicates, and the error bars represent standard deviations

The time courses of cellulase production from Avicel and SECS are presented in Fig. 3a. The Avicel induced a slightly higher filter paper activity (FPA) than the SECS in the cellulase production by *T. reesei* Rut-C30. On Day 5, the FPAs induced by the Avicel and SECS were 2.87 ± 0.29 and 2.45 ± 0.36 FPIU/mL, respectively. The FPAs then decreased because the *T. reesei* Rut-C30 entered a declining phase, which was in line with previous results [19, 20]. Thus, the cellulases harvested on Day 5 were applied for self-enzymatic hydrolysis in the context of on-site cellulase production [13–15], i.e., the cellulase induced by Avicel used for the enzymatic hydrolysis of Avicel, because this was advantageous over using the cellulases induced by other substrates or commercial cellulases [15, 21]. The results of the enzymatic saccharification of Avicel and SECS are shown in Fig. 3b. Enzymatic hydrolysis of SECS was found to have a higher yield than that of Avicel. The former produced 39.73 ± 0.95 g/L glucose from 100 g/L SECS containing 53.2 g/L glucan, and the latter produced 28.31 ± 1.17 g/L glucose from 50 g/L Avicel. The resultant hydrolysates were subsequently fermented by the engineered *S. cerevisiae* strains, LGA-1 and LGA-C, for d-glucaric acid production. As shown in Fig. 3c, the time courses showed similar patterns. Though the enzymatic hydrolysate of SECS fermented by LGA-1 led to the highest d-glucaric acid titer of 4.92 ± 0.24 g/L after 7 d of fermentation, the differences were not highly distinguishable, and the enzymatic hydrolysate of Avicel had a lower concentration of glucose.

Overall, LGA-1 produced 3.74 ± 0.31 g/L d-glucaric acid and LGA-C produced 3.93 ± 0.38 g/L d-glucaric acid from 50 g/L Avicel. LGA-1 produced 4.92 ± 0.24 g/L d-glucaric acid and LGA-C produced 4.63 ± 0.52 g/L d-glucaric acid from 100 g/L SECS.

### Simultaneous saccharification and fermentation (SSF)

**Fig. 4:**
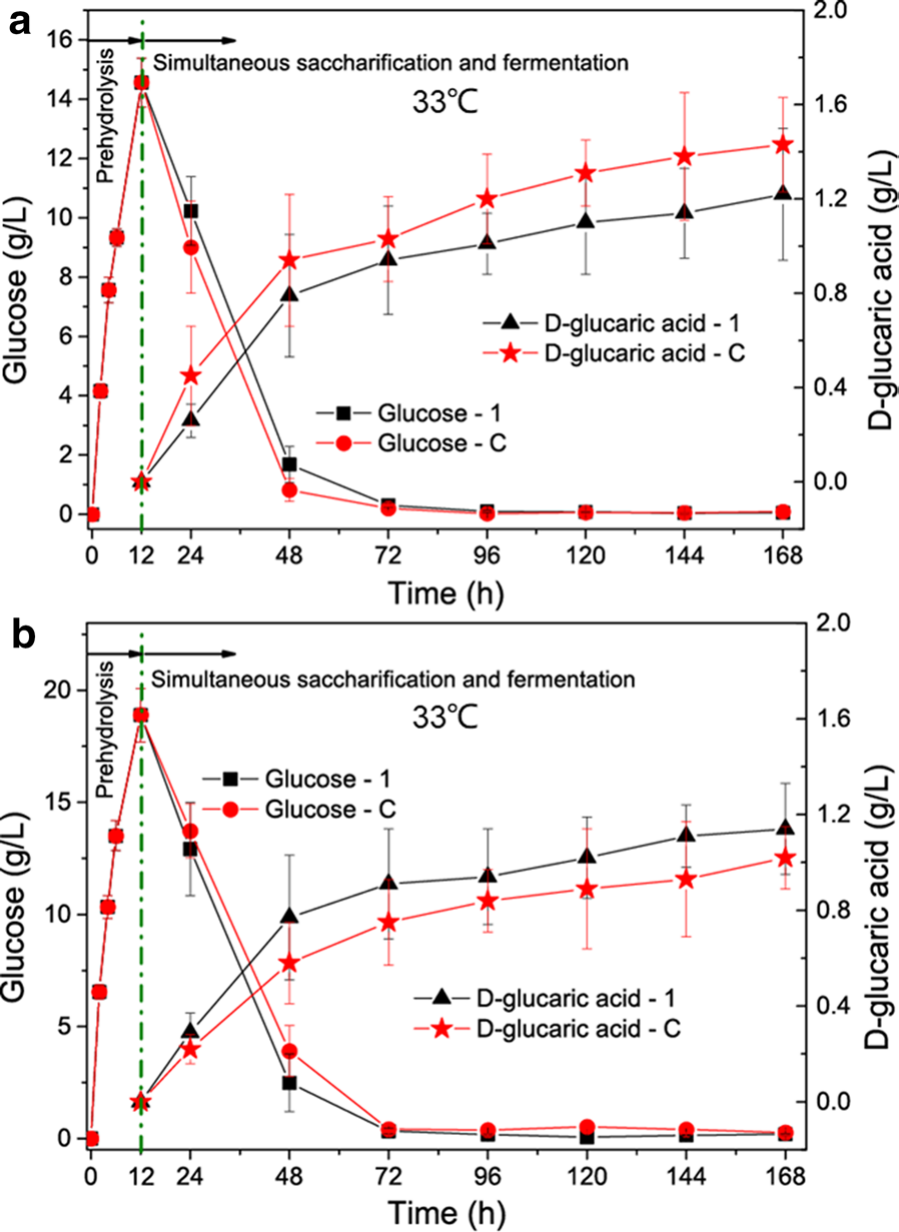
Simultaneous saccharification and fermentation of **a** Avicel and **b** steam-exploded corn stover (SECS) at 33 °C from 12 to 168 h after enzymatic pre-hydrolysis at 50 °C for 12 h. LGA-1 and LGA-C are the engineered *S. cerevisiae* strains capable of producing d-glucaric acid. The data shown here are average values of at least three biological replicates, and the error bars represent standard deviations

Three temperatures, 30 °C, 33 °C, and 36 °C, were tested to study the effect on SSF and the results are shown in Additional files 2, 3: Fig. S2 and S3, Fig. 4, respectively. It was found that 33 °C was the most suitable temperature for SSF, leading to the highest concentrations of d-glucaric acid. This was the same as the SSF for bioethanol production, where 33 °C was also the most suitable temperature [11].

The time courses of SSF for d-glucaric acid production from Avicel and SECS are shown Fig. 4a and b, respectively. They displayed a similar pattern in which glucose dropped rapidly after LGA-1 or LGA-C was inoculated. After 48 h, the glucose concentration was reduced to an extremely low level close to zero. The titers of d-glucaric acid increased quickly after 12 h, which was the time point of *S. cerevisiae* inoculation, and it reached a plateau after 72 h. LGA-1 and LGA-C showed opposite results in the SSF for producing d-glucaric acid. The former produced a higher titer of d-glucaric acid than the latter from Avicel, but the former produced a lower titer from SECS. However, the differences were small.

After 7 d, LGA-1 produced 1.22 ± 0.28 g/L d-glucaric acid and LGA-C produced 1.43 ± 0.20 g/L d-glucaric acid from 50 g/L Avicel. LGA-1 produced 1.14 ± 0.19 g/L d-glucaric acid and LGA-C produced 1.02 ± 0.13 g/L d-glucaric acid from 100 g/L SECS.

### Consolidated bioprocessing (CBP)

**Fig. 5:**
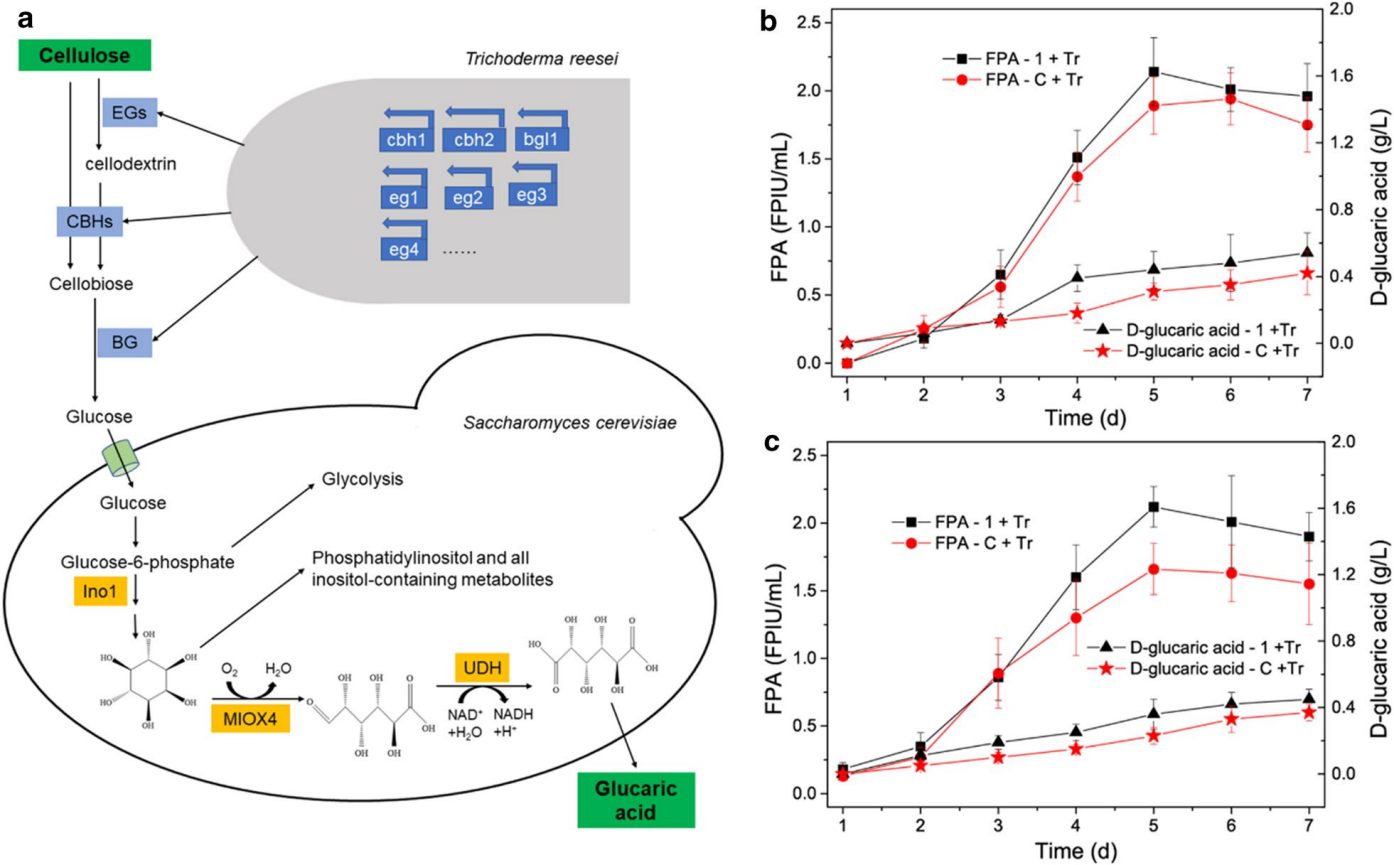
**a** Diagram of CBP of cellulose for d-glucaric acid production by the microbial consortium consisting of *T. reesei* Rut-C30 and *S. cerevisiae* LGA-1. EGs, endoglucanases; CBHs, cellobiohydrolases; BG, β-glucosidase; Ino1, *myo*-inositol-1-phosphate synthase; MIOX4, *myo*-inositol oxygenase; UDH, uronate dehydrogenase. **b** FPAs and concentrations of d-glucaric acid during CBP of Avicel. **c** FPAs and concentrations of d-glucaric acid during CBP of SECS. The labels “1” and “C” in legends stand for *S. cerevisiae* LGA-1 and LGA-C, respectively, and “Tr” stands for *T. reesei* Rut-C30. The data shown here are average values of at least three biological replicates, and the error bars represent standard deviations

CBP is a desirable method for d-glucaric acid production from lignocellulose because both cellulase production by *T. reesei* and d-glucaric acid production by *S. cerevisiae* are aerobic, unlike CBP for bioethanol production where an anaerobic condition should be created for *S. cerevisiae* [12]. The CBP operation was much simpler. CBP for d-glucaric acid production from lignocellulose by *T. reesei* and *S. cerevisiae* is illustrated in Fig. 5a. The microbial consortium composed of *T. reesei* Rut-C30 and the engineered *S. cerevisiae* could achieve the bioconversion of lignocellulose to d-glucaric acid in one step.

The time courses of CBPs from Avicel and SECS are shown in Fig. 5b and c, respectively. The microbial consortium of *T. reesei* Rut-C30 and *S. cerevisiae* LGA-1 produced 0.54 ± 0.12 g/L d-glucaric acid from 15 g/L Avicel and 0.45 ± 0.06 g/L d-glucaric acid from 15 g/L SECS after 7 d of fermentation. Somewhat lower concentrations of d-glucaric acid were produced when *S. cerevisiae* LGA-C was used. Thus, *S. cerevisiae* LGA-1 was more desirable in the CBP for d-glucaric acid production. An increase in substrate loading did not lead to higher concentrations of d-glucaric acid, as shown in Additional file 4: Fig. S4. The optimal substrate loading in CBP for d-glucaric acid production (15 g/L) was lower than that in CBP for bioethanol production (17.5 g/L) [12]. This result indicates the low efficiency of the microbial consortium comprising *T. reesei* Rut-C30 and *S. cerevisiae* LGA-1 in the CBP of lignocellulose to d-glucaric acid.

Further work was carried out to improve the efficiency. The effect of the ratio of *T. reesei* to *S. cerevisiae* on CBP was investigated and the results are shown in Additional file 5: Fig. S5. Among the inoculum ratios of *T. reesei* to *S. cerevisiae* (1:1, 1:3, 1:5, 5:1, and 3:1), 1:1 was found to be the best for CBP. Changing the ratio caused a lower d-glucaric acid titer and was unable to improve the efficiency. The effect of the delay time of *S. cerevisiae* inoculation on CBP was also studied, and the results are presented in Additional file 6: Fig. S6. Among the delay times of *S. cerevisiae* inoculation (0, 24, and 48 h), 0 h (i.e., *T. reesei* and *S. cerevisiae* were inoculated simultaneously) was found to be the most suitable.

**Fig. 6:**
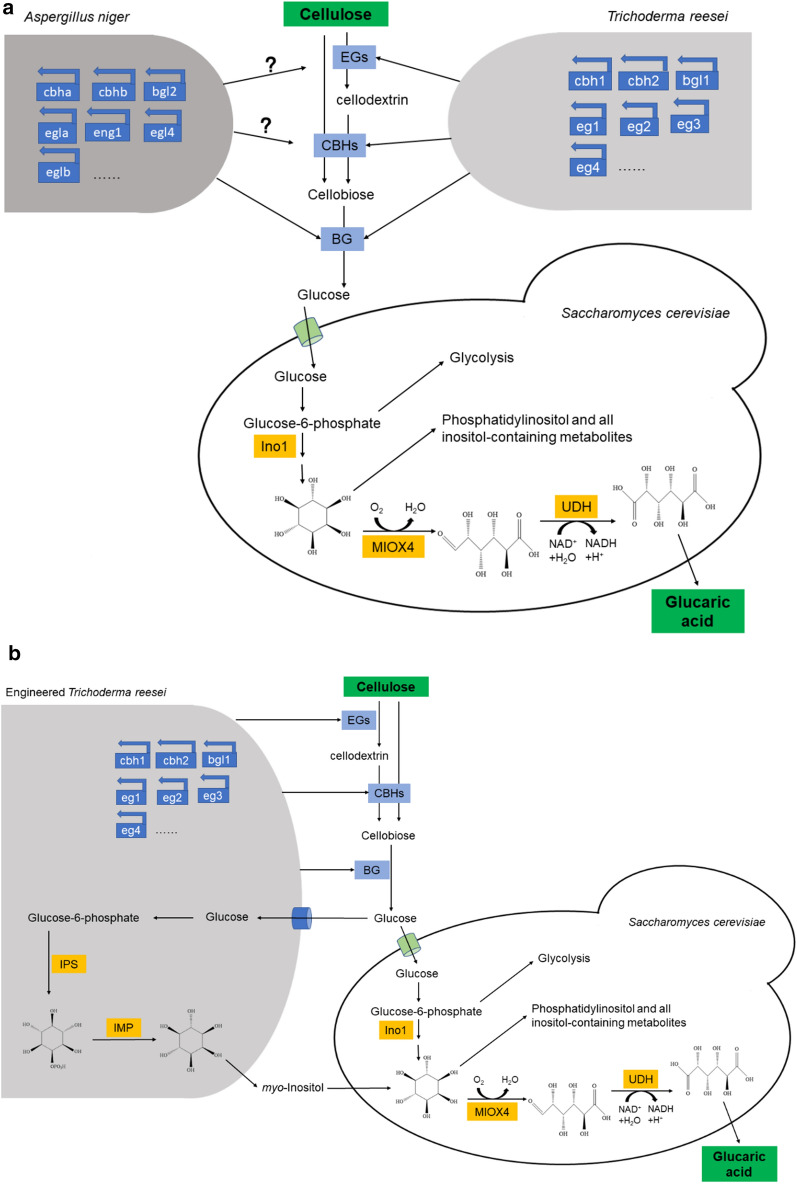

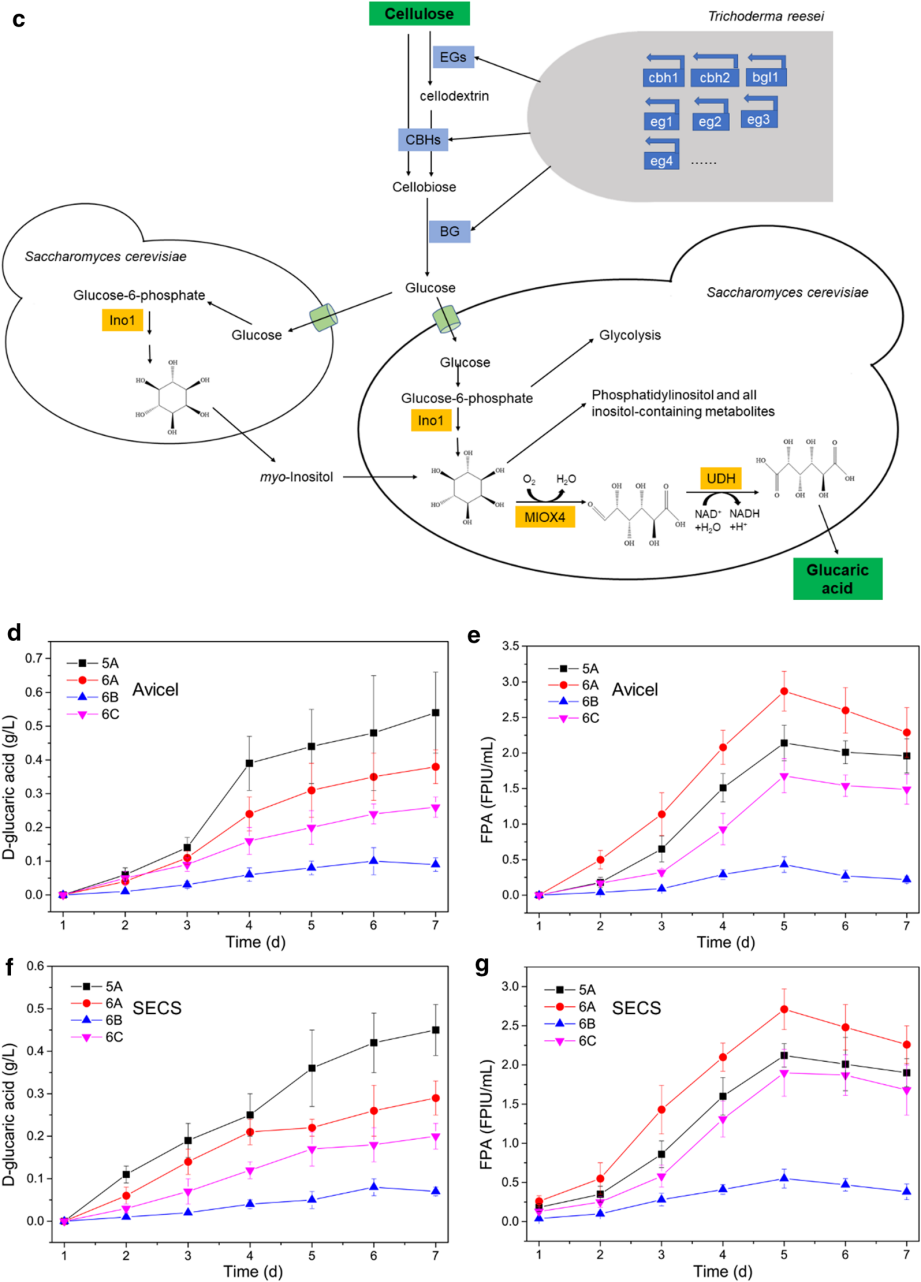
**a** Diagram of CBP of cellulose for d-glucaric acid production by the microbial consortium consisting of *T. reesei* Rut-C30, *A. niger* CICC2103 and *S. cerevisiae* LGA-1. **b** Diagram of CBP of cellulose for d-glucaric acid production by the microbial consortium consisting of the engineered *T. reesei* and *S. cerevisiae* LGA-1. **c** Diagram of CBP of cellulose for d-glucaric acid production by the microbial consortium consisting of *T. reesei* Rut-C30, *S. cerevisiae Δopi1* and *S. cerevisiae* LGA-1. Concentrations of **d** d-glucaric acid and **e** FPAs during CBP of Avicel. Concentrations of **f** d-glucaric acid and **g** FPAs during CBP of SECS. The labels “5A”, “6A”, “6B”, and “6C” in the legends of **d**, **e**, **f**, and **g** represent the CBPs illustrated in Figs. 5a, 6a, b, and c, respectively. The data shown here are average values of at least three biological replicates, and the error bars represent standard deviations

In light of the previous finding that the mixed culture of *T. reesei* and *A. niger* could enhance cellulase production [20, 22], *A. niger* was introduced into the microbial consortium to improve the efficiency of lignocellulose degradation. The ratio of *T. reesei* to *A. niger* to *S. cerevisiae* was 5:1:5 and the ratio of the total inocula to the fermentation medium was 10% (v/v). The results are presented in Fig. 6a, d, e, f and g, and it was found that the microbial consortium composed of *T. reesei*, *A. niger* and *S. cerevisiae* produced lower concentrations of d-glucaric acid than the microbial consortium consisting of *T. reesei* and *A. niger*, though the former produced a higher FPA. This suggested that the microbial consortium of *T. reesei*, *A. niger* and *S. cerevisiae* was not suitable for the CBP of lignocellulose for d-glucaric acid production.

Given that Gupta et al. [5] found that *myo*-inositol availability was the rate-limiting step in the engineered *S. cerevisiae* expressing the *miox4* gene from *A. thaliana*, we adopted the following strategies to increase the *myo*-inositol availability. We engineered *T. reesei* Rut-C30 to excrete *myo*-inositol by expressing inositol-3-phosphate synthase (*ips*, GenBank: L23520.1) and inositol monophosphatase (*imp*, GenBank: CP029160.1) from *S. cerevisiae*. However, this was proven to be unsuccessful because engineering *T. reesei* to produce *myo*-inositol made it no longer potent in cellulase production, and the concentration of *myo*-inositol produced by the engineered *T. reesei* was negligible (Additional file 7: Fig. S7). The results in Fig. 6b, d, e, f, and g indicate that this strategy was not feasible for improving d-glucaric acid production by CBP of lignocellulose.

We next turned our focus to the engineered *S. cerevisiae* with the *opi1* gene being knocked out, whose myo-inositol accumulation was improved (Additional file 8: Fig. S8), i.e., the parental strains of LGA-1 and LGA-C. *S. cerevisiae* Δ*opi1* was added to the microbial consortium to attempt to improve the efficiency of CBP for d-glucaric acid production. The inoculum ratio of *T. reesei* to *S. cerevisiae* Δ*opi1* to *S. cerevisiae* LGA-1 was 2:1:1, and the total volume of the inocula was 10% (v/v) of the fermentation medium. Figure 6c, d, e, f, and g shows that this strategy was inferior to the strategy shown in Fig. 5a.

Overall, the microbial consortium of *T. reesei* Rut-C30 and *S. cerevisiae* LGA-1 was the most efficient CBP for d-glucaric acid production from lignocellulose. Neither the addition of the third strain nor the metabolic engineering of *T. reesei* for enhanced *myo*-inositol availability was successful in improving the efficiency of CBP.

### Comparison of different biorefinery processes for d-glucaric acid production

The parameters of different biorefinery processes for d-glucaric production from Avicel and SECS, including SHF, SSF and CBP, are listed in Tables 1 and 2, respectively. The fermentation processes from pure glucose with or without *myo*-inositol supplementation were used as references for comparison. The fermentation of glucose supplemented with *myo*-inositol had the highest d-glucaric acid titer. Next was the d-glucaric acid production from glucose or from the hydrolysates of SECS and Avicel containing glucose. SHF had a d-glucaric acid titer close to that from pure glucose without *myo*-inositol supplementation. SSF had lower d-glucaric acid titers from Avicel and SECS than those of SHF and much lower yields than those of SHF. This suggests that SSF was not a desirable approach for d-glucaric acid production from lignocellulose due to its low efficiency.

**Table 1:** Details of the bioprocesses from Avicel to d-glucaric acid

	SHF^a^	SSF^b^	CBP^c^	Glu^d^	G + I^e^
Substrate	Avicel	Avicel	Avicel	Glucose	Glucose and *myo*-inositol
Avicel for bioprocesses (g)	50	50	15	0	0
Glucose (g)	28.31	N.A	N.A	30	30
*myo*-Inositol (g)	0	0	0	0	10.8
Cellulase dosage (FPIU/g glucan)	25	25	0		0
Total cellulase loading (FPIU)	1250	1250	0	0	0
Avicel for cellulase production (g)	13.07	13.07	0	0	0
Total Avicel (g)	63.07	63.07	15	0	0
d-Glucaric acid (g)	3.74	1.22	0.54	4.52	9.53
Yield (g d-glucaric acid/g total Avicel)	0.0593	0.0193	0.0360	N.A	N.A

Glu, glucose

G + I, glucose and *myo*-inositol

N.A., not applicable

^a^SHF, separated saccharification and fermentation by *S. cerevisiae* LGA-1

^b^SSF, simultaneous saccharification and fermentation by *S. cerevisiae* LGA-1

^c^CBP, consolidated bioprocessing by *T. reesei* Rut-C30 and *S. cerevisiae* LGA-1

^d^Fed-batch fermentation of glucose by *S. cerevisiae* LGA-1

^e^Fed-batch fermentation of glucose and *myo*-inositol by *S. cerevisiae* LGA-1

**Table 2:** Details of the bioprocesses from steam-exploded corn stover (SECS) to d-glucaric acid

	SHF^a^	SSF^b^	CBP^c^	Glu^d^	G + I^e^
Substrate	SECS	SECS	SECS	Glucose	Glucose and *myo*-inositol
SECS for bioprocesses (g)	100	100	15	0	
Glucose (g)	39.73	N.A	N.A	30	30
*myo*-Inositol (g)	0	0	0	0	10.8
Cellulase dosage (FPIU/g glucan)	25	25	0	0	0
Total cellulase loading (FPIU)	2500	2500	0	0	0
SECS for cellulase production (g)	30.61	30.61	0	0	0
Total SECS (g)	130.61	130.61	15	0	0
d-Glucaric acid (g)	4.92	1.14	0.45	4.52	9.53
Yield (g d-glucaric acid/g total SECS)	0.0377	0.0087	0.0300	N.A	N.A

Glu, glucose

G + I, glucose and *myo*-inositol

N.A., not applicable

^a^SHF, separated saccharification and fermentation by *S. cerevisiae* LGA-1

^b^SSF, simultaneous saccharification and fermentation by *S. cerevisiae* LGA-1

^c^CBP, consolidated bioprocessing by *T. reesei* Rut-C30 and *S. cerevisiae* LGA-1

^d^Fed-batch fermentation of glucose by *S. cerevisiae* LGA-1

^e^Fed-batch fermentation of glucose and *myo*-inositol by *S. cerevisiae* LGA-1

Though CBP had a relatively low d-glucaric acid titer, it had considerably higher yields from Avicel and SECS. This was because a low substrate loading was used. The yields of the CBP were 0.0360 g d-glucaric acid/g Avicel and 0.0300 g d-glucaric acid/g SECS, which were higher than those of SSF but slightly lower than those of SHF (0.0593 g d-glucaric acid/g Avicel and 0.0377 g d-glucaric acid/g SECS). Moreover, CBP has the fewest single-unit operations and the greatest potential for cost reduction [10, 12, 23] because both the cellulase production by *T. reesei* Rut-C30 and d-glucaric acid production by *S. cerevisiae* LGA-1 during the CBP of lignocellulose by the microbial consortium were aerobic. This reduces the difficulty and complexity of constructing and operating the microbial consortium [12]. Therefore, this is a highly promising approach for d-glucaric production from lignocellulose.

## Discussion

The strategy of constructing a *S. cerevisiae* strain for d-glucaric acid production at a high titer was applied to a different yeast strain, *S. cerevisiae* INVSc1, in this work [3]. Two engineered strains with high titers of d-glucaric acid, LGA-1 and LGA-C, were obtained. LGA-C with the episomal expression of *miox4* and *udh* had a higher concentration of d-glucaric acid than LGA-1 with the integrated expression when supplementing *myo*-inositol (Fig. 2b). However, no difference was evident when using glucose as the sole carbon source. This may have been because episomal expression was more efficient in transforming *myo*-inositol into d-glucaric acid than integrated expression when abundant *myo*-inositol was available or the recombinant plasmid with a high copy number led to more MIOX4 and UDH than the integration into Ty loci. It seems that both LGA-1 and LGA-C acted as whole-cell catalysts when the fermentation was supplemented with *myo*-inositol. In the fed-batch fermentation supplemented with *myo*-inositol, both LGA-1 and LGA-C produced a record-breaking titer of d-glucaric acid, 9.53 ± 0.46 g/L, and LGA-C produced 11.21 ± 0.63 g/L d-glucaric acid, which was much higher than previously reported values on *S. cerevisiae* [3, 5] and *E. coli* [2, 4, 7, 24]. It is noteworthy that higher d-glucaric acid was produced in this work than in the work of Chen et al. [3], though we adopted the same strategy. This was likely because a different *S. cerevisiae* strain was used. Different strains introduce various differences, including different physiological statuses, efficiencies of foreign gene expression, and different *myo*-inositol availabilities.

The high titer of d-glucaric acid produced by *S. cerevisiae* LGA-C demonstrated the great potential of *S. cerevisiae* INVSc1 in d-glucaric acid production, although episomal expression was problematic because of its genetic instability [3]. The higher titer of d-glucaric acid produced from glucose and *myo*-inositol than that from glucose suggested that the *myo*-inositol availability was still the rate-liming step for d-glucaric acid production in the engineered *S. cerevisiae*. Moreover, *myo*-inositol is a valuable chemical in the industry [17, 18]. Therefore, the production of d-glucaric acid from *myo*-inositol was not as economically competitive as direct production from glucose. To enhance the d-glucaric acid production from glucose, further work should be done to improve the biosynthesis pathway efficiency of d-glucaric acid in *S. cerevisiae*.

As one of the top value-added chemicals from biomass [1], there have been no reports on d-glucaric acid production from lignocellulose, the most abundant renewable on earth. The biorefinery processes, SHF, SSF, and CBP (Fig. 1), were conducted using the engineered *S. cerevisiae* strains, LGA-1 and LGA-C. Two substrates were used in these biorefinery processes, Avicel and SECS. The former was pure cellulose, which is often used as model substrate, and the latter was corn stover, which is abundant in China but is always improperly treated, causing serious air pollution [21]. The results of SHF (Fig. 3) showed that Avicel induced more cellulase but had lower glucose concentrations. Overall, both the d-glucaric acid titer and yield of SECS were comparable to those of Avicel (Tables 1 and 2). Moreover, the results of SHF were close to fermentation from pure glucose. These results indicated that corn stover is a suitable feedstock for d-glucaric acid production and that the engineered *S. cerevisiae* could be applied to the biorefinery of lignocellulose for d-glucaric production.

SSF was proven to be less successful in this study, leading to the lowest d-glucaric acid titers and yields from Avicel and SECS (Tables 1 and 2). The success of SSF was less because SSF was not authentic and it encompassed two phases enzymatic pre-hydrolysis and SSF. Only the former phase had the highest hydrolysis efficiency. After entering SSF, the temperature was decreased to the appropriate range for *S. cerevisiae* in which the enzymatic hydrolysis rate was reduced. When the SSF temperature was increased for better enzymatic hydrolysis, the fermentation by *S. cerevisiae* was weakened (Fig. 4, Additional files 2, 3: Fig. S2 and S3). The hurdle of SSF is the gap between the optimal temperature for enzymatic hydrolysis by *T. reesei* cellulase and that for fermentation by *S. cerevisiae*. SSF for bioethanol production is confronted with the same challenge [11].

Enlightened by the previous research on CBP for bioethanol production [12], we constructed the same microbial consortium of *T. reesei* and *S. cerevisiae* and applied it to the CBP of lignocellulose for d-glucaric acid production. The results (Fig. 5, Tables 1 and 2) are promising because the consortium produced relatively high yields, demonstrating its efficiency. However, there is still room to increase the efficiency of CBP by *T. reesei* and *S. cerevisiae*. The ratio of *T. reesei* to *S. cerevisiae* was investigated, and 1:1 was the most suitable (Additional file 5: Fig. S5). This is reasonable because decreasing the ratio weakened the lignocellulose degradation capability, and increasing the ratio attenuated the fermentation by the engineered *S. cerevisiae* for d-glucaric acid production. We then delayed the inoculation of *S. cerevisiae*, resulting in reduced d-glucaric acid production. Simultaneous inoculations of *T. reesei* and *S. cerevisiae* were the most suitable. This was because delaying *S. cerevisiae* inoculation allowed *T. reesei* to consume more substrate, and less was left for d-glucaric acid production.

The cellulase from *T. reesei* Rut-C30 was deficient in β-glucosidase and previous research proved that the mixed culture of *T. reesei* and *A. niger* could improve the cellulase production and the cellulose deconstruction capability [20, 22, 25]. We introduced *A. niger* into the microbial consortium, and the cellulase production during CBP was enhanced (Fig. 6). On the contrary, the d-glucaric acid concentration was lowered. It is plausible that *A. niger* consumed resources but did no contributed to d-glucaric acid production in the team of three members, therefore impairing the efficiency of CBP. A similar situation occurred when we introduced *S. cerevisiae opi1Δ* into the microbial consortium to increase the *myo*-inositol availability (Fig. 6 and Additional file 8: S8). We then engineered *T. reesei* Rut-C30 to allow it to provide *myo*-inositol during CBP. However, once *T. reesei* was engineered, its cellulase production capability was crippled (Additional file 7: Fig. S7). This made the microbial consortium weaker in lignocellulose degradation, negatively affecting the efficiency of CBP for d-glucaric acid production from lignocellulose (Fig. 6). The failure in the metabolic engineering of *T. reesei* discouraged the further efforts of *T. reesei* to enable direct production of d-glucaric acid from lignocellulose. Even if successful, it was nearly impossible to obtain a super strain that was simultaneously good at cellulase production and d-glucaric acid production. A microbial cell factory would be faced with a significant metabolic dilemma when performing these two production processes simultaneously [26]. Thus, the artificial microbial consortium of *T. reesei* Rut-C30 (cellulase specialist) and *S. cerevisiae* LGA-1 (d-glucaric acid specialist) was an excellent team with “chemistry” in CBP.

In the long run, direct production of d-glucaric acid from lignocellulose is advantageous over that from glucose and *myo*-inositol. Due to the highest integration of single-unit operations, the utmost simplicity of bioprocess control, and the cheapest substrate, CBP is promising for the production of d-glucaric acid from lignocellulose. The relatively high d-glucaric acid titer and yield from this work proved that CBP of lignocellulose by the artificial microbial consortium of *T. reesei* and *S. cerevisiae* deserves extensive and in-depth research. The most important research task is to improve the efficiency of the CBP by the microbial consortium, which is still low at the current stage. We are engineering *T. reesei* to enhance enzymatic hydrolysis and *S. cerevisiae* to improve the biosynthesis of d-glucaric acid. The ultimate aim is to realize a distributive and collaborative push-and-pull strategy to promote the CBP by the microbial consortium of *T. reesei* and *S. cerevisiae* for d-glucaric acid production from lignocellulose.

## Conclusions

The biosynthesis pathway of d-glucaric acid production was constructed in *S. cerevisiae* INVSc1, whose *opi1* was knocked out, by expressing *miox4* from *A. thaliana* and *udh* from *P. syringae*. Two high-titer d-glucaric acid-producing strains were obtained, LGA-1 and LGA-C. Both produced record-breaking titers of d-glucaric acid, indicating that *S. cerevisiae* INVSc1 was an excellent host. However, these high d-glucaric acid titers were achieved by *myo*-inositol supplementation, which is not preferable to using lignocellulose. SHF, SSF, and CBP were studied, and the CBP using an artificial microbial consortium of *T. reesei* Rut-C30 and the engineered *S. cerevisiae* is a promising approach with a relatively high titer and yield. The microbial consortium was redesigned for higher efficiency, but the two members, *T. reesei* Rut-C30 and *S. cerevisiae* LGA-1, were proven to be the best teammates for CBP. Further work should be done to improve the efficiency of this microbial consortium for d-glucaric acid production from lignocellulose.

## Methods

### Plasmids and their construction

The *miox4* gene encoding MIOX4 in *A. thaliana* [27] and the *udh* gene encoding UDH in *P. syringae* [28] were codon-optimized for the expression in *S. cerevisiae* and synthesized by Sangon Biotech (Shanghai, China). These two genes were then ligated to the plasmid of pY26-GPD-TEF, purchased from Miaoling Bioscience & Technology Co., Ltd. (Wuhan, China), using *Bgl*II/*Not*I and *Eco*RI/*Xho*I, to generate pY26-miox4-udh (Additional file 9: Table S1). The recombinant vectors carrying *miox4* and *udh* under the control of the promoters *Pgpd* and *Ptef*, respectively, were used for the subsequent transformation of *S. cerevisiae*.

The plasmid pUG6 from Miaoling Bioscience & Technology Co., Ltd. (Wuhan, China) was used as the template for constructing the knock out cassette loxP-Kan-loxP by polymerase chain reaction (PCR) using knock-OPI1F/R primers (Additional file 10: Table S2). The plasmid pSH47 from Miaoling Bioscience & Technology Co., Ltd. (Wuhan, China) provided Cre recombinase for the self-recombination of the knock out cassette.

The plasmid pCAMBIA1300 [19, 29] was used as the backbone for constructing the recombinant vector pCA-Pcbh1-ips-Tcbh1 (Additional file 9: Table S1), where the gene *ips* (GenBank: L23520.1) encoding *myo*-inositol-1-phosphate synthase from *S. cerevisiae* [2] was codon-optimized according to the codon preference of *T. reesei* and expressed under the control of the strong promoter *Pcbh1* in *T. reesei* to improve the *myo*-inositol production.

### Strains and media

All strains used in this work, including the starting strains and the engineered strains, are listed in Additional file 9: Table S1.

LB medium, containing 1 g/L tryptone, 0.5 g/L yeast extract, and 1 g/L NaCl, was used to culture *E. coli* and *Agrobacterium tumefaciens* cells after autoclaving at 121 °C for 20 min. YPD medium was used to culture *S. cerevisiae* cells, which had the following composition: 10 g/L yeast extract, 20 g/L peptone, and 20 g/L glucose. For the selective YPD medium, geneticin G418 was added to a specific concentration after being autoclaved and cooled. Solid media were prepared by adding 2 g/L agar before autoclaving.

The seed medium for *T. reesei* strains was composed of 10 g/L glucose, 1 g/L peptone, 5 mL of Mandels nutrient salt solution [30], 2.5 mL of citrate buffer (1 mol/L), 0.05 mL of Mandels trace element solution [30], and 0.1 g/L Tween 80. The seed medium was autoclaved at 121 °C for 20 min. The fermentation medium for cellulase production by *T. reesei* comprised 15 g/L Avicel or 30 g/L pretreated lignocellulose (dry biomass), 1 g/L glucose, 6 g/L (NH_4_)_2_SO_4_, 2.0 g/L KH_2_PO_4_, 0.3 g/L CaCl_2_, 0.3 g/L MgSO_4_, 0.005 g/L FeSO_4_, 0.0016 g/L MnSO_4_, 0.0014 g/L ZnSO_4_ and 0.0037 g/L CoCl_2_. The initial pH was adjusted to 4.8 with citrate buffer. The fermentation medium was autoclaved at 121 °C for 30 min.

All chemicals except lignocellulosic materials were purchased from Sinopharm Chemical Reagent Co. Ltd. (Shanghai, China). The media for SSF and CBP are described below.

### Genetic engineering of *S. cerevisiae*

The loxP-Kan-loxP cassette amplified from pUG6 by PCR using the primers knock-OPI1F/R (Additional file 10: Table S2) was transformed into competent cells of *S. cerevisiae* INVSc1 prepared with the Li-Ac method [31] to knock out *opi1*. The *KanMX* gene disruption cassette was cured by a homologous recombination of the loxP sites mediated by Cre recombinase expressed by the plasmid pSH47. pSH47 was lost as the host cells were cultivated continuously, resulting in the *S. cerevisiae* INVSc1 *opi1Δ* strain, which was used as the host for constructing the biosynthesis pathway for d-glucaric acid production.

There are two ways to express foreign genes in *S. cerevisiae*, episomal or integrative. For the episomal expression, the recombinant vector pY26-miox4-udh was directly transformed into the competent cells of *S. cerevisiae* INVSc1 *opi1Δ*. For the integrative expression, the expression cassettes were spliced by overlap PCR using delta1 and delta2 and integrated into Ty loci [3, 32]. First, delta1 and delta2 were amplified from the *S. cerevisiae* genome using the primers delta1-F/delta1-R and delta2-F/delta2-R (Additional file 10: Table S2), respectively, and the *miox4* and *udh* expression cassettes were amplified from the plasmid pY26-miox4-udh using the MIXO4-F/R and UDH-F/R primers, respectively. Second, the fragments d1-M-F and L-U-d2 were generated by overlap PCR from delta1 and the MIXO4 expression cassette using the primers delta1-F and FURA3-R and from the UDH expression cassette and delta2 using the primers LURA-F/delta2-R, respectively. Finally, the whole fragment was produced by overlap PCR of the fragments d1-M-F and L-U-d2 using the primers delta1-F and delta2-R. The final product was purified with Cycle-Pure Kit 200 (Omega Bio-tek, Georgia, USA) and then used for the transformation of the competent cells of *S. cerevisiae* INVSc1 *opi1Δ*.

### Genetic engineering of *T. reesei*

The recombinant plasmid pCA-Pcbh1-ips-Tcbh1 was transformed into *T. reesei* Rut-C30 by the method of *A. tumefaciens*-mediated transformation (AMT) [33]. The potential *T. reesei* transformants were then selected by two rounds of screening, the first PDA (potato dextrose agar) plates with added hygromycin B and the second Avicel plates as described above [19, 29]. The fast-growing *T. reesei* transformants selected by the two rounds of screening were tested in *myo*-inositol production and used in the microbial consortium to increase *myo*-inositol availability.

### Fermentation for d-glucaric acid production by engineered *S. cerevisiae*

Fermentation was carried out in 250-mL Erlenmeyer flasks with a working volume of 50 mL of YPD medium with or without 10.8 g/L (60 mM) *myo*-inositol. Before inoculation into fermentation medium, *S. cerevisiae* strains were precultured in 5 mL of YPD medium with 10.8 g/L (60 mM) *myo*-inositol in 50-mL Erlenmeyer flasks at 30 °C while shaking at 250 rpm until an optical density at 600 nm (OD_600_) of ~ 5 was achieved. The cells were then collected and inoculated into fermentation medium with or without 10.8 g/L (60 mM) *myo*-inositol to an OD_600_ of 0.1. Fermentation was implemented at 30 °C while shaking at 250 rpm.

Fed-batch fermentation was conducted under conditions similar to those of the batch fermentation mentioned above, except that 5 g/L glucose was supplemented twice during the fermentation process, at 24 and 48 h.

### Separated hydrolysis and fermentation (SHF)

In the SHF (Fig. 1), the cellulase produced by *T. reesei* Rut-C30 from Avicel or steam-exploded corn stover (SECS) [14, 34, 35] was applied for self-enzymatic hydrolysis through the so-called on-site cellulase production [13–15, 35]. *T. reesei* Rut-C30 was precultured in the seed medium for 36 h, and then the seed was collected and inoculated into the fermentation medium for cellulase production. The fermentation broth containing crude cellulase was directly used in the enzymatic hydrolysis of Avicel or SECS [14, 29], which was conducted in 250-mL Erlenmeyer flasks with 50 mL of reaction mixture containing 2.5 mL of 1 M citrate buffer solution (for final pH 4.8), 50 g/L Avicel or 100 g/L SECS (dry material), 25 FPIU/g of glucan from the cellulase harvested after 5 d of fermentation, and an amount of water to reach a total volume of 50 mL. Enzymatic hydrolysis was conducted at 50 °C while shaking at 140 rpm for 48 h. The resulting enzymatic hydrolysates containing fermentable sugars were used to prepare the fermentation medium supplemented with the same nutrients as in the YPD medium except for glucose. The following operations were the same as those described in the section describing the fermentation for d-glucaric acid production by the engineered *S. cerevisiae*.

### Simultaneous saccharification and fermentation (SSF)

In the SSF (Fig. 1), cellulase prepared in the same way as that for the SHF was used in the enzymatic pre-hydrolysis of Avicel or SECS for 12 h. The pre-hydrolysis of SSF was performed in 250-mL Erlenmeyer flasks with 50 mL of SSF reaction mixture containing 50 g/L Avicel or 100 g/L SECS (dry material), 25 FPIU/g of glucan from the cellulase harvested after 5 d of fermentation, 6 g/L (NH_4_)_2_SO_4_, 2.0 g/L KH_2_PO_4_, 0.3 g/L MgSO_4_∙7H_2_O, 0.3 g/L CaCl_2_∙2H_2_O, 0.1 g/L Tween 80, 10 g/L peptone, 5 g/L yeast extract, and an amount of water to reach a total volume of 50 mL. Before adding cellulase, the initial pH was adjusted to 4.8 with citrate buffer and the reaction mixture was autoclaved at 121 °C for 30 min. After cellulase was added, the reaction mixture was incubated at 50 °C while shaking at 140 rpm for 12 h. The temperature was decreased to 33 °C (or as specified in the main text when studying its effect on SSF), and the shaking was increased to 250 rpm. The precultured *S. cerevisiae* with an OD_600_ of ~ 5 was inoculated into the reaction mixture for SSF.

### Consolidated bioprocessing (CBP)

The medium for CBP had a following composition: 15 g/L (or as specified in the main text when studying its effect on CBP) Avicel or SECS (dry material), 1 g/L peptone, 1 g/L yeast extract, 10% (v/v) Mandels nutrient salt solution [30], 0.1% (v/v) trace element solution [30], 5%(v/v) citrate buffer (1 mol/L), and 0.1 g/L Tween 80. CBP medium was autoclaved at 121 °C for 30 min. *T. reesei* was precultured in the seed medium at 30 °C for 36 h, and *S. cerevisiae* was precultured in YPD medium at 30 °C to an OD_600_ of ~ 5. *T. reesei* was then inoculated into CBP medium and the inoculation of *S. cerevisiae* was implemented immediately or delayed (or as specified in the main text (Results on CBP) when studying its effect on CBP). The inoculum ratio of *T. reesei* to *S. cerevisiae* was 1:1 (or as specified in the main text when studying its effect on CBP). When other strains or species were inoculated, the relevant information is specified in the main text. The total inocula were 10% (v/v) of the fermentation medium. CBP was carried out in 250-mL Erlenmeyer flasks with 50 mL of medium at 30 °C while shaking at 180 rpm. *A. niger* was precultured for 48 h using the same method as that used for *T. reesei* if needed.

### Analytical methods

The FPA of cellulase, representing the total enzymatic activity, was assayed with the method standardized by the International Union of Pure and Applied Chemistry (IUPAC) [36]. It was used to quantify the total quantity of the reducing sugar produced from 50 mg of Whatman No.1 filter paper (1 cm × 6 cm strip) by cellulase for 60 min. One International Unit of FPA (FPIU) was defined as the amount of cellulase needed to produce 1 μmol of reducing sugar in 1 min.

β-Glucosidase activity (BGA) was determined using the standard method [36] with a slight modification, i.e., the substrate ρNPG (ρ-nitrophenyl-β-d-1,4-glucopyranoside) (Sigma-Aldrich, St. Louis, MO, USA). The amount of ρ-nitrophenol produced from ρNPG by β-glucosidase within 10 min was assayed using a spectrophotometer at a wavelength of 400 nm. One International Unit of BGA (IU) was defined as the amount of β-glucosidase required to produce 1 μmol of ρ-nitrophenol from ρNPG in 1 min.

Cellobiohydrolase activity (CBA) was assayed according to the method modified from the FPA measurement method [36]. Microcrystalline cellulose PH101 purchased from Sigma-Aldrich (St. Louis, MO, USA) in the form of a 1% (w/v) suspension was used as the substrate for the reaction with a duration of 30 min. One Unit (1 U) of CBA was defined as the amount of enzyme required to produce 1 mg of reducing sugar in 1 h.

High-performance liquid chromatography (HPLC) was used to analyze and quantify the d-glucaric acid, *myo*-inositol, and sugar contents. A Shimadzu Prominence LC-20A system equipped with Bio-Rad Aminex HPX-87H (300 mm × 7.8 mm) column was used. When d-glucaric acid content was determined, an ultraviolet (UV) detector was employed to detect the eluate; when *myo*-inositol and sugar contents were determined, a refractive index (RI) detector was employed. Sulfuric acid (5 mmol/L) was used as the mobile phase with a flow rate of 0.6 mL/min. The column temperature was maintained at 50 °C. Sample loading for each injection was 10 μL.

Liquid chromatography–mass spectrometry (LC–MS) was used to characterize and identify d-glucaric acid, where a Shimadzu L-30A and an AB Sciex Triple TOF 5600 were employed. The column was a Shimadzu Shim-pack XR-ODS 100 L × 2.0. The mobile phases, with a flow rate of 0.15 mL/min, were an aqueous solution containing 1 mmol/L ammonium formate and 1% (v/v) formic acid (mobile phase A) and an acetonitrile solution containing 1 mmol/L ammonium formate (mobile phase B). Mobile phase B (B for short hereinafter), was used as the eluent, and the elution procedure was as follows: 0–12 min, 30% B; 12–30 min, 30%–65% B; 30–31 min, 65%–95% B; 31–35 min, 95% B; 35–36 min, 95%–30% B; 36–40 min, 30% B. Sample loading for each injection was 5 μL. Negative electrospray ionization mode was chosen to ionize the samples. The flow rate of atomizing gas (N_2_) was 1.5 L/min. The temperature for CDL and HB was 200 °C. The scanning scope (m/z) ranged from 150 to 300.

## Supplementary Information

**Additional file 1: Fig. S1.** Results of liquid chromatography–mass spectrometry (LC–MS) analysis. (A) LC and (B) MS graphs of the fermentation broth of *S. cerevisiae* LGA-1 after 7 d of fed-batch fermentation in YPD medium supplemented with 10 g/L glucose and 10.8 g/L *myo*-inositol. (C) MS graph of the standard of d-glucaric acid

**Additional file 2: Fig. S2.** Simultaneous saccharification and fermentation of (A) Avicel and (B) steam-exploded corn stover (SECS) at 30 °C from 12 to 168 h after enzymatic pre-hydrolysis at 50 °C for 12 h. LGA-1 and LGA-C are the engineered *S. cerevisiae* strains capable of producing d-glucaric acid. The data shown here are average values of at least three biological replicates, and the error bars represent standard deviations.

**Additional file 3: Fig. S3.** Simultaneous saccharification and fermentation of (A) Avicel and (B) SECS at 36 °C from 12 to 168 h after enzymatic pre-hydrolysis at 50 °C for 12 h. LGA-1 and LGA-C are the engineered *S. cerevisiae* strains capable of producing d-glucaric acid. The data shown here are average values of at least three biological replicates, and the error bars represent standard deviations.

**Additional file 4: Fig. S4.** Time courses of CBPs of 15, 17.5 and 20 g/L Avicel or SECS for d-glucaric acid production by *S. cerevisiae* LGA-1. (A) Concentrations of d-glucaric acid and yields during CBP of Avicel. (B) FPAs during CBP of Avicel. (C) Concentrations of d-glucaric acid and yields during CBP of SECS. (D) FPAs during CBP of SECS. The data shown here are average values of at least three biological replicates, and the error bars represent standard deviations.

**Additional file 5: Fig. S5.** Effects of the inoculum ratio of *T. reesei* Rut-C30 to *S. cerevisiae* LGA-1 on the CBPs of (A and B) 15 g/L Avicel and (C and D) SECS for d-glucaric acid production. (A) Concentrations of d-glucaric acid during CBP of Avicel. (B) FPAs during CBP of Avicel. (C) Concentrations of d-glucaric acid during CBP of SECS. (D) FPAs during CBP of SECS. The data shown here are average values of at least three biological replicates, and the error bars represent standard deviations.

**Additional file 6: Fig. S6.** Effects of the delay time of *S. cerevisiae* LGA-1 inoculation on the CBPs of (A and B) 15 g/L Avicel and (C and D) SECS by the microbial consortium of *T. reesei* Rut-C30 and *S. cerevisiae* LGA-1 for d-glucaric acid production. (A) Concentrations of d-glucaric acid during CBP of Avicel. (B) FPAs during CBP of Avicel. (C) Concentrations of d-glucaric acid during CBP of SECS. (D) FPAs during CBP of SECS. The data shown here are average values of at least three biological replicates, and the error bars represent standard deviations.

**Additional file 7: Fig. S7.** (A) FPAs and CBAs during fermentation on 15 g/L Avicel by *T. reesei* Rut-C30 and the engineered *T. reesei* for *myo*-inositol production. (B) Concentrations of *myo*-inositol after 5 d of fermentation. The data shown here are average values of at least three biological replicates, and the error bars represent standard deviations.

**Additional file 8: Fig. S8.** Concentrations of *myo*-inositol produced by *S. cerevisiae* strains after 5 d of fermentation on YPD medium. INVSc1 was the starting strain, and INVSc1 + Δopi1 was the engineered *S. cerevisiae* whose *opi1* was knocked out. The data shown here are average values of at least three biological replicates, and the error bars represent standard deviations.

**Additional file 9: Table S1.** Plasmids and strains used in this study.

**Additional file 10: Table S2.** Oligomers used in this study.

## Data Availability

Not applicable.

## Abbreviations

AMT: *Agrobacterium* *tumefaciens*-Mediated transformation
BGA: Beta-glucosidase activity
CBP: Consolidated bioprocessing
FPA: Filter paper activity
HPLC: High-performance liquid chromatography
Ino1: Myo-inositol-1-phosphate synthase
LC–MS: Liquid chromatography–mass spectrometry
MIOX: *myo*-Inositol oxygenase
PDA: Potato dextrose agar
RI: Refractive index
SECS: Steam-exploded corn stover
SHF: Separated hydrolysis and fermentation
SSF: Simultaneous saccharification and fermentation
UDH: Uronate dehydrogenase
UV: Ultraviolet
