# Supplementary material for: Consolidated bioprocessing of lignocellulose for production of glucaric acid by an artificial microbial consortium
Source: Biotechnol Biofuels. 2021 Apr 30;14:110. doi: 10.1186/s13068-021-01961-7 (PMC8086319; doi:10.1186/s13068-021-01961-7)
Supplement: Supplementary file 1 — Additional file 1: Fig. S1. Results of liquid chromatography–mass spectrometry (LC–MS) analysis. (A) LC and (B) MS graphs of the fermentation broth of S. cerevisiae LGA-1 after 7 d of fed-batch fermentation in YPD medium supplemented with 10 g/L glucose and 10.8 g/L myo-inositol. (C) MS graph of the standard of d-glucaric acid [file 13068_2021_1961_MOESM1_ESM.docx]

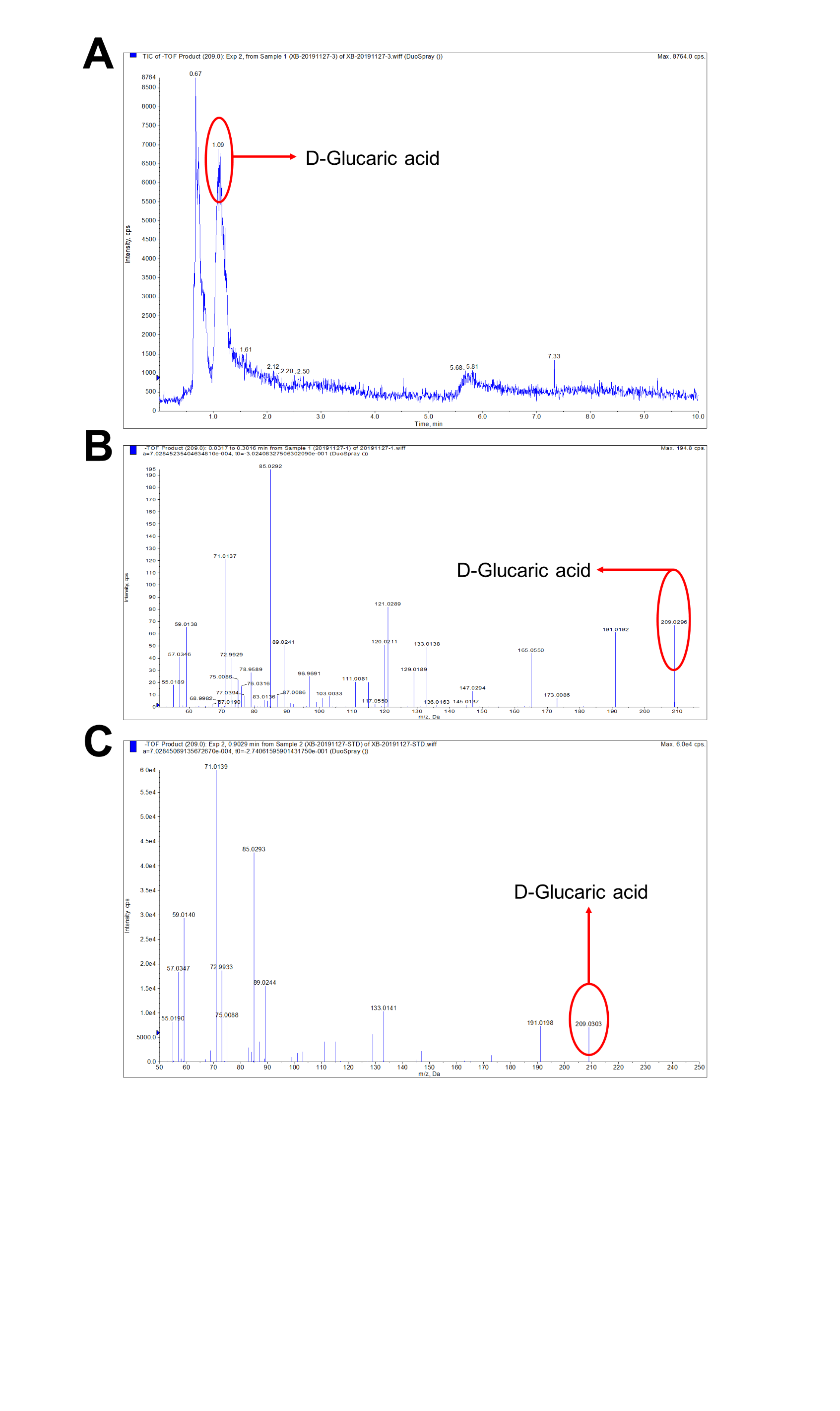


Fig. S1. Results of liquid chromatography–mass spectrometry (LC-MS) analysis. (A) LC and (B) MS graphs of the fermentation broth of *S. cerevisiae* LGA-1 after 7 d of fed-batch fermentation in YPD medium supplemented with 10 g/L glucose and 10.8 g/L *myo*-inositol. (C) MS graph of the standard of D-glucaric acid.
