# Supplementary material for: Consolidated bioprocessing of lignocellulose for production of glucaric acid by an artificial microbial consortium
Source: Biotechnol Biofuels. 2021 Apr 30;14:110. doi: 10.1186/s13068-021-01961-7 (PMC8086319; doi:10.1186/s13068-021-01961-7)
Supplement: Supplementary file 3 — Additional file 3: Fig. S3. Simultaneous saccharification and fermentation of (A) Avicel and (B) SECS at 36 °C from 12 to 168 h after enzymatic pre-hydrolysis at 50 °C for 12 h. LGA-1 and LGA-C are the engineered S. cerevisiae strains capable of producing d-glucaric acid. The data shown here are average values of at least three biological replicates, and the error bars represent standard deviations. [file 13068_2021_1961_MOESM3_ESM.docx]

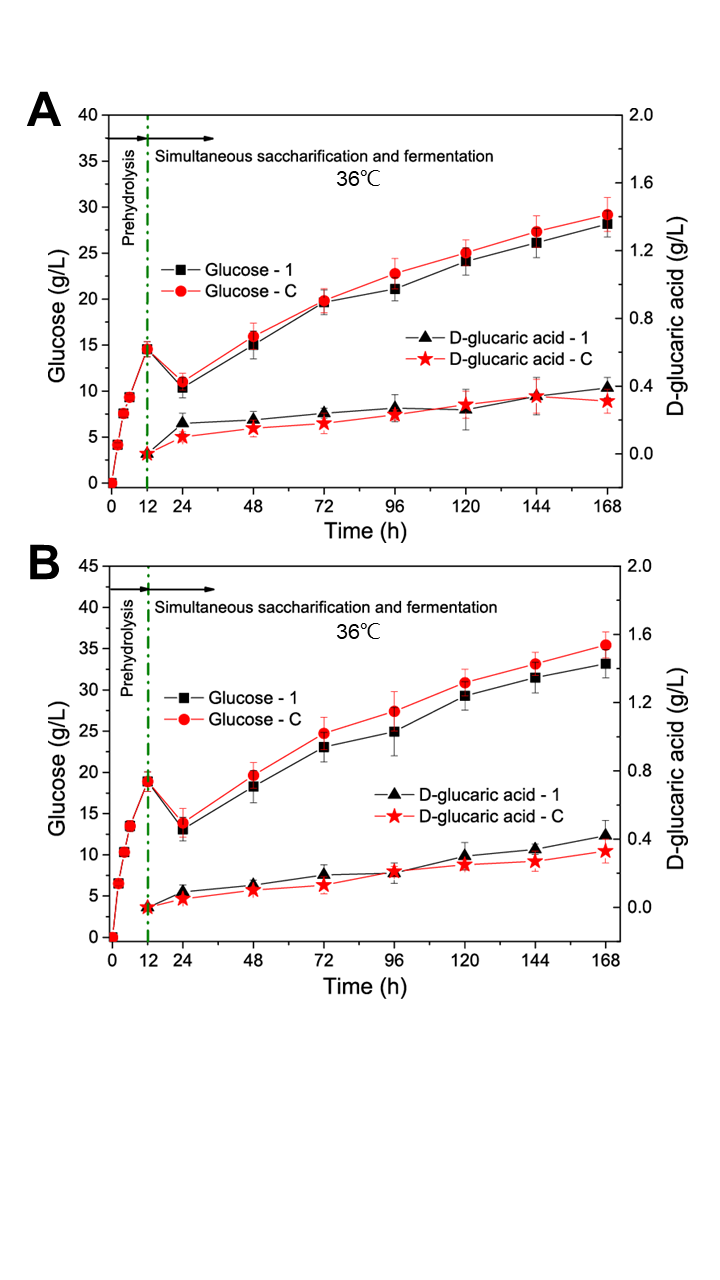


Fig. S3. Simultaneous saccharification and fermentation of (A) Avicel and (B) SECS at 36°C from 12 to 168 h after enzymatic pre-hydrolysis at 50°C for 12 h. LGA-1 and LGA-C are the engineered *S. cerevisiae* strains capable of producing D-glucaric acid. The data shown here are average values of at least three biological replicates, and the error bars represent standard deviations.
