# Supplementary material for: Consolidated bioprocessing of lignocellulose for production of glucaric acid by an artificial microbial consortium
Source: Biotechnol Biofuels. 2021 Apr 30;14:110. doi: 10.1186/s13068-021-01961-7 (PMC8086319; doi:10.1186/s13068-021-01961-7)
Supplement: Supplementary file 4 — Additional file 4: Fig. S4. Time courses of CBPs of 15, 17.5 and 20 g/L Avicel or SECS for d-glucaric acid production by S. cerevisiae LGA-1. (A) Concentrations of d-glucaric acid and yields during CBP of Avicel. (B) FPAs during CBP of Avicel. (C) Concentrations of d-glucaric acid and yields during CBP of SECS. (D) FPAs during CBP of SECS. The data shown here are average values of at least three biological replicates, and the error bars represent standard deviations. [file 13068_2021_1961_MOESM4_ESM.docx]

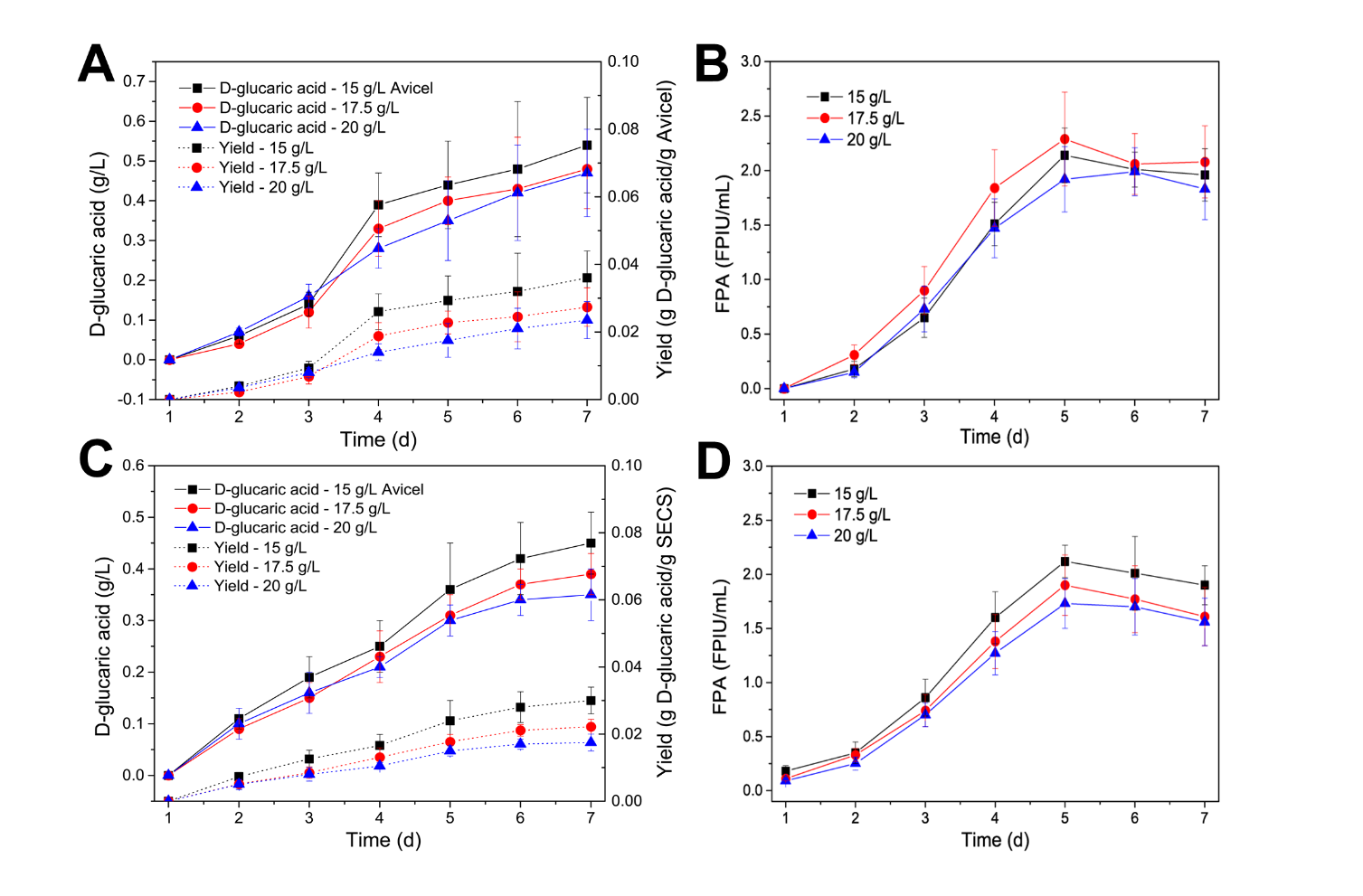


Fig. S4. Time courses of CBPs of 15, 17.5 and 20 g/L Avicel or SECS for D-glucaric acid production by *S. cerevisiae* LGA-1. (A) Concentrations of D-glucaric acid and yields during CBP of Avicel. (B) FPAs during CBP of Avicel. (C) Concentrations of D-glucaric acid and yields during CBP of SECS. (D) FPAs during CBP of SECS. The data shown here are average values of at least three biological replicates, and the error bars represent standard deviations.
