# Supplementary material for: Consolidated bioprocessing of lignocellulose for production of glucaric acid by an artificial microbial consortium
Source: Biotechnol Biofuels. 2021 Apr 30;14:110. doi: 10.1186/s13068-021-01961-7 (PMC8086319; doi:10.1186/s13068-021-01961-7)
Supplement: Supplementary file 5 — Additional file 5: Fig. S5. Effects of the inoculum ratio of T. reesei Rut-C30 to S. cerevisiae LGA-1 on the CBPs of (A and B) 15 g/L Avicel and (C and D) SECS for d-glucaric acid production. (A) Concentrations of d-glucaric acid during CBP of Avicel. (B) FPAs during CBP of Avicel. (C) Concentrations of d-glucaric acid during CBP of SECS. (D) FPAs during CBP of SECS. The data shown here are average values of at least three biological replicates, and the error bars represent standard deviations. [file 13068_2021_1961_MOESM5_ESM.docx]

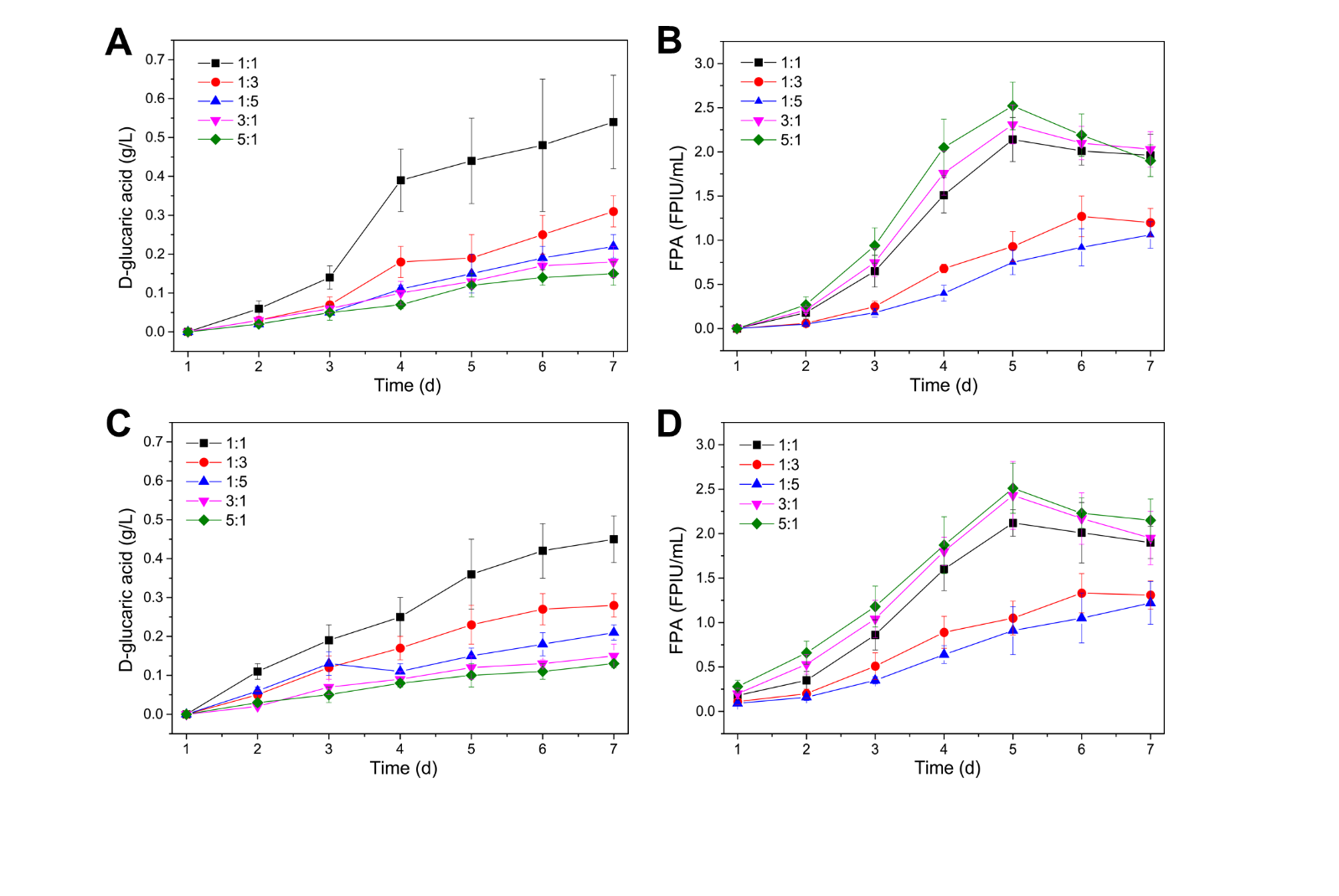


Fig. S5. Effects of the inoculum ratio of *T. reesei* Rut-C30 to *S. cerevisiae* LGA-1 on the CBPs of (A and B) 15 g/L Avicel and (C and D) SECS for D-glucaric acid production. (A) Concentrations of D-glucaric acid during CBP of Avicel. (B) FPAs during CBP of Avicel. (C) Concentrations of D-glucaric acid during CBP of SECS. (D) FPAs during CBP of SECS. The data shown here are average values of at least three biological replicates, and the error bars represent standard deviations.
