# Supplementary material for: Consolidated bioprocessing of lignocellulose for production of glucaric acid by an artificial microbial consortium
Source: Biotechnol Biofuels. 2021 Apr 30;14:110. doi: 10.1186/s13068-021-01961-7 (PMC8086319; doi:10.1186/s13068-021-01961-7)
Supplement: Supplementary file 7 — Additional file 7: Fig. S7. (A) FPAs and CBAs during fermentation on 15 g/L Avicel by T. reesei Rut-C30 and the engineered T. reesei for myo-inositol production. (B) Concentrations of myo-inositol after 5 d of fermentation. The data shown here are average values of at least three biological replicates, and the error bars represent standard deviations. [file 13068_2021_1961_MOESM7_ESM.docx]

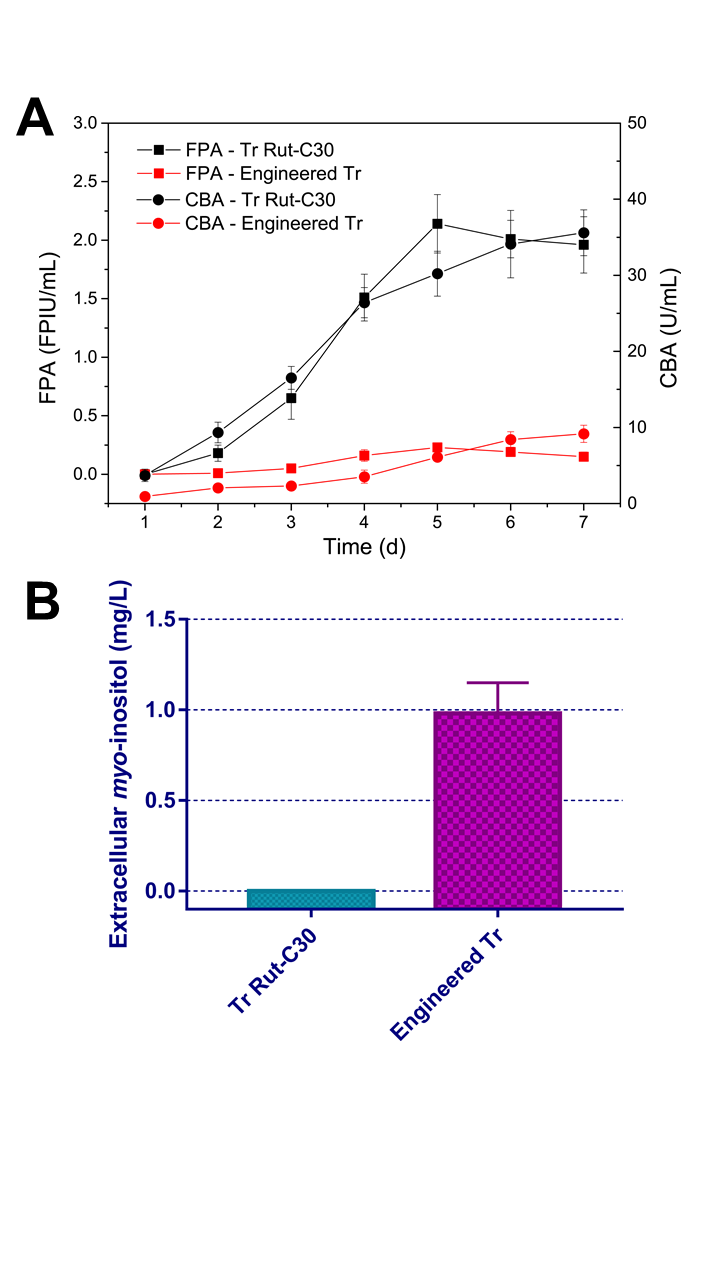


Fig. S7. (A) FPAs and CBAs during fermentation on 15 g/L Avicel by *T. reesei* Rut-C30 and the engineered *T. reesei* for *myo*-inositol production. (B) Concentrations of *myo*-inositol after 5 d of fermentation. The data shown here are average values of at least three biological replicates, and the error bars represent standard deviations.
