# Supplementary material for: Consolidated bioprocessing of lignocellulose for production of glucaric acid by an artificial microbial consortium
Source: Biotechnol Biofuels. 2021 Apr 30;14:110. doi: 10.1186/s13068-021-01961-7 (PMC8086319; doi:10.1186/s13068-021-01961-7)
Supplement: Supplementary file 8 — Additional file 8: Fig. S8. Concentrations of myo-inositol produced by S. cerevisiae strains after 5 d of fermentation on YPD medium. INVSc1 was the starting strain, and INVSc1 + Δopi1 was the engineered S. cerevisiae whose opi1 was knocked out. The data shown here are average values of at least three biological replicates, and the error bars represent standard deviations. [file 13068_2021_1961_MOESM8_ESM.docx]

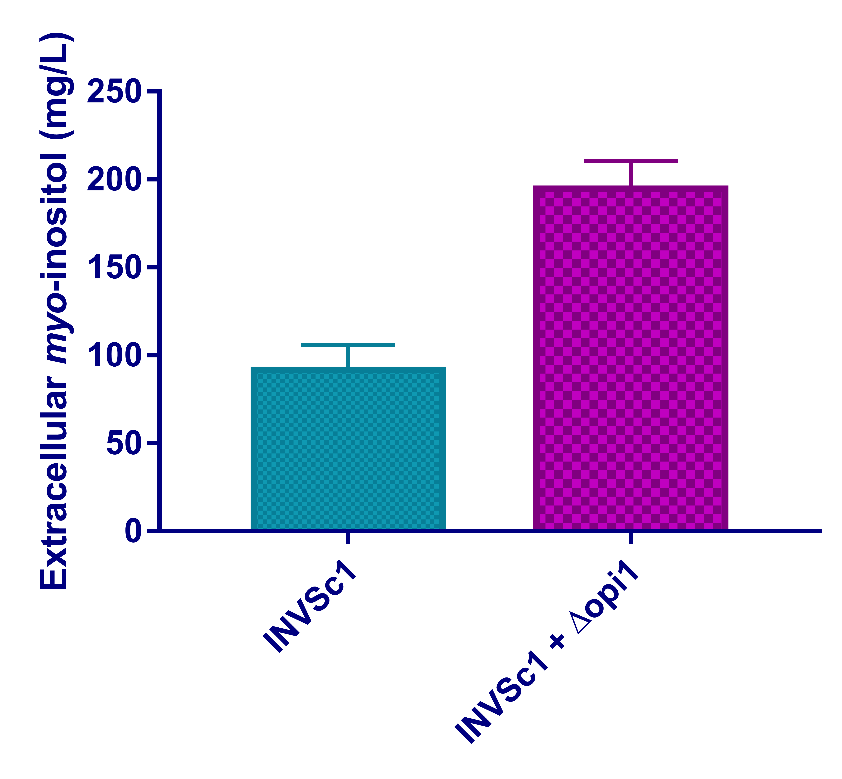


Fig. S8. Concentrations of *myo*-inositol produced by *S. cerevisiae* strains after 5 d of fermentation on YPD medium. INVSc1 was the starting strain, and INVSc1 + Δopi1 was the engineered *S. cerevisiae* whose *opi1* was knocked out. The data shown here are average values of at least three biological replicates, and the error bars represent standard deviations.
